# Mechanisms linking teenage mothers’ educational attainment with self-reported health at age 50

**DOI:** 10.1186/s12905-020-01150-y

**Published:** 2021-01-06

**Authors:** Julie Maslowsky, C. Emily Hendrick, Haley Stritzel

**Affiliations:** 1 Department of Kinesiology and Health Education, University of Texas at Austin, 2109 San Jacinto D3700, Austin, TX 78712 USA; 2 Department of Population Health, Dell Medical School, University of Texas at Austin, Austin, TX USA; 3 Population Research Center, University of Texas at Austin, Austin, TX USA; 4 School of Community Health Sciences, University of Nevada Reno, Reno, NV USA

**Keywords:** Teenage childbearing, Educational attainment, Self-reported health, Life course theory

## Abstract

**Background:**

Early childbearing is associated with adverse health and well-being throughout the life course for women in the United States. As education continues to be a modifiable social determinant of health after a young woman gives birth, the association of increased educational attainment with long-term health for women who begin childbearing as teenagers is worthy of investigation.

**Methods:**

Data are from 301 mothers in the National Longitudinal Survey of Youth 1979 who gave birth prior to age 19. We estimated path models to assess women’s incomes, partner characteristics, and health behaviors at age 40 as mediators of the relationship between their educational attainment and self-rated general health at age 50.

**Results:**

After accounting for observed background factors that select women into early childbearing and lower educational attainment, higher levels of education (high school diploma and GED attainment vs. no degree) were indirectly associated with higher self-rated health at age 50 via higher participant income at age 40.

**Conclusions:**

As education is a social determinant of health that is amenable to intervention after a teen gives birth, our results are supportive of higher educational attainment as a potential pathway to improving long-term health outcomes of women who begin childbearing early.

## Background

Teenage childbearing is associated with adverse health and socioeconomic disadvantage for women and their children in the United States [1]. On the one hand, the disadvantage faced by teenage mothers may be due to the background characteristics that selected them into early childbearing [2]. Alternatively, the experience of teenage motherhood itself—e.g., truncated education, time out of the labour force, discrimination by peers—may also contribute to worse outcomes [3]. Although most research focuses on short-term outcomes for women and their children, a small body of evidence demonstrates that health concerns associated with early childbearing last throughout the life course for women [4, 5]. What remains unclear, however, are the mechanisms that promote long-term health of women who begin childbearing during the teenage years. Attaining additional education after the birth of a child during the teen years is a modifiable social influence factor that may support women’s health and well-being across the life course [6]. In order to inform effective programming and policies to support women’s health and wellbeing across the life course, a better understanding of these mechanisms is needed.

### Women’s age at childbearing and their health throughout the life course

Life course theory purports that life experiences, and the timing of life experiences, influence health and social development trajectories [7]. Women who begin childbearing as teenagers in the United States have worse health, earn less education, and are more likely to live in poverty and receive public assistance in adulthood than women who delay childbearing until their 20 s or later [8–10]. In addition, women who begin childbearing during the teenage years continue to exhibit relatively poor physical and mental health, including higher mortality rates, into mid-life [4, 5, 11–13].

Despite decades of community- and school-based programming in the United States aimed toward supporting pregnant and parenting teens, such as hospital-based Teen Tot programs, home visitation programs, and school-based child care [14, 15], how to improve their long-term health is not yet well understood. Once a young woman gives birth, the socio-economic background from which she has emerged may be immutable. In other words, we can no longer change the social-selection factors that selected her into teenage childbearing. However, women with early childbearing, on average, earn low levels of education, and education continues to be a modifiable social determinant of health after a young woman gives birth.

### Educational attainment of women who begin childbearing during the teenage years and implications for long-term health

Educational attainment is a major predictor of health across the lifespan. Even after accounting for selection effects, the amount of formal schooling a woman completes is a strong predictor of her health and well-being across her life [16]. As only half of women who give birth as teenagers graduate from high school (compared to 90% of women who delay childbearing beyond the teenage years [17], truncated education may be responsible for some of the downstream health and socioeconomic disadvantage associated with early childbearing. Regardless of whether the increased risk for adverse outcomes later in life is due to truncated education, or to social selection factors occurring before young women’s first births, increasing educational attainment may be a promising strategy for improving health later in life. Further, in the United States, those who are least likely to attain high levels of education demonstrate the greatest educational returns for health outcomes later in life [18]. Most research focuses on the short-term educational outcomes [19–21] of teenage mothers, but there is a notable dearth of research examining the association of educational attainment and health later in the life course. One exception, using data from the Midlife Development in the US study, did not find education to significantly mediate the association between teenage childbearing and mid-life health and health behaviours [11]. However, this study provided suggestive evidence that teenage childbearing is associated with women’s health via effects on labour force participation, income, and spouse’s socioeconomic status, all of which may be affected by educational attainment. As such, additional longitudinal research is needed to determine to what extent education may be associated with the long-term health of women who begin childbearing early, via mechanisms occurring later in life.

### Mechanisms by which education may be associated with the long-term health of women who begin childbearing during the teenage years

**Fig. 1:**
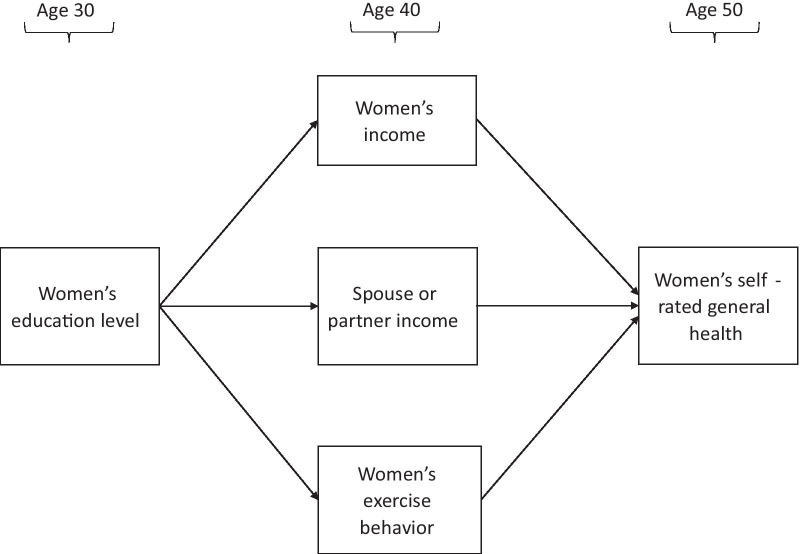
Conceptual model of pathways from women’s educational attainment to later life health

Three major theoretical perspectives provide insight into the mechanisms through which greater educational attainment is linked with better health. First, the theory of fundamental causes proposes that education provides access to flexible resources such as greater income, knowledge, prestige, power, and social connections, all of which can be leveraged to improve health [22]. Second, human capital theory emphasizes the health returns to the skills, abilities, and knowledge developed by greater educational attainment [23]. Third, credentialing theory expands upon human capital theory to consider how high school and/or college degrees signal an individual’s skills, abilities, and knowledge to important social actors [24]. The social response from people such as employers or potential spouses can facilitate access to more highly-educated social networks and desirable jobs, thus leading to improved socioeconomic outcomes [25]. In turn, increased socioeconomic attainment can improve health through multiple mechanisms. Figure 1 illustrates the conceptual pathways tested in the current study by which women’s greater educational attainment may be associated with long-term health.

First, higher incomes associated with higher levels of education allow for increased access to a multitude of health-promoting resources in adulthood including healthier living environments, higher quality food, and higher quality health care [26, 27]. Also, higher levels of education may alter women’s social networks and expose them to romantic partners with higher earning potential, thereby increasing their household income level, providing emotional and practical support for parenting, and influencing healthy behaviours in adulthood [28]. Finally, higher levels of education may directly influence women’s health behaviours by providing a learned appreciation for the importance of health behaviours, such as engaging in regular physical activity. Although participating in regular physical activity may have direct positive impacts on women’s health later in life, such as reducing risk for obesity and stress-related diseases and increasing cardiovascular health, it can also serve as a marker of women’s health behaviours in adulthood [29]. Participating in regular physical activity often corresponds with other positive health behaviours such as eating healthy diets and avoiding smoking [30–32]. Thus, in the present study, women’s exercise behaviours at age 40 served as a proxy for their general health behaviours in adulthood.

### The current study

We sought to identify to what extent, and through what mechanisms, higher levels of educational attainment (GED or high school diploma vs. no degree) were associated with higher self-rated general health at age 50 for women who began childbearing as teenagers. Importantly, we focused on education attained after the birth of their first child during the teen years, to highlight the potential impact of this modifiable factor for teenage mothers. Drawing from life course theory, we hypothesized women earning higher levels of education by age 30 would experience higher levels of personal income, spouse/partner income, and more positive health behaviours at age 40 that would in turn be associated with better self-reported health at age 50.

## Methods

### Data

Data are from Waves 1 (1979) through 26 (2014) of the National Longitudinal Survey of Youth 1979 (NLSY79) [24]. Participants were aged 14–22 at Wave 1 and aged 49–58 at Wave 26. The analytic sample included 301 women gave birth as teenagers, but did not have their first birth prior to their Wave 1 interview, had completed fewer than 12 years of school by their Wave 1 interview, and completed the Health Module at age 50. Due to the analytic modelling employed, NLSY79 sampling weights were not used per NLSY79 recommendations [33]. Table 1 presents descriptive characteristics of the analytic sample (*N* = 301). This study was deemed not to be human subjects research by the Institutional Review Board of the sponsoring university.

**Table 1:** Unweighted descriptive characteristics of analytic sample (*N* = 301)

	Mean or proportion	SD
Self-reported general health at age 50 (1–5)	3.03	1.11
First birth prior to age 19	1.00	
Degree type attained by age 30		
No degree	0.36	
High school diploma or GED	0.64	
Potential mediators (age 40)		
Participant income (divided by $10,000)	1.71	1.70
Spouse/partner income (logged)	3.88	5.07
Frequency of vigorous exercise behaviour (0–4)	2.08	1.72
Background characteristics (age 14–22)		
Mothers' highest grade completed (0–19)	9.34	2.92
Enrolled in school at Wave 1	0.85	
AFQT aptitude percentile score at Wave 1 (0–100)	24.97	20.43
Highest grade expected to earn at Wave 1 (1–18)	12.65	2.14
Race/ethnicity		
Black/Non-hispanic	0.46	
Hispanic	0.26	
Non-black/Non-hispanic	0.29	
Self-esteem score at Wave 2 (7–30)	20.74	3.70
Number of siblings at Wave 1 (0–19)	4.55	2.86

*GED* General Education Development/High School Equivalency Certificate

### Measures

#### Self-rated general health at age 50

The dependent variable was a self-rated measure of general health: “In general, would you say your health is excellent, very good, good, fair, or poor?” coded 1–5 with 1 = “poor,” 5 = “excellent.” Self-rated general health measures are widely used in public health research to provide an overall assessment of individuals’ current health status [34]. Lower levels of self-rated general health are associated with physical and mental health conditions and symptoms [35, 36] and are salient predictors of mortality [37].

#### Educational attainment by age 30

The primary independent variable was women’s educational attainment by age 30. Each year, participants were asked to report their highest degree completed. Few women who gave birth as teenagers earned college degrees (*n* = 4). In addition, few women (*n* = 7) reported receiving a GED but not a high school diploma by age 30. Therefore, participants who completed a GED or completed additional years of schooling beyond high school diploma were combined with those who had earned a high school diploma to form a single category representing earning at least a high school degree or GED by age 30. Those reporting receiving neither a GED, high school degree, nor college degree by age 30 were categorized as having “no degree.” Due to interview scheduling and missed interviews, not all women reported educational outcomes at age 30. For these women, we used educational attainment reported at the interview immediately prior to when they turned 30. Consequently, we also controlled for the age at which women reported their educational outcomes (aged 25–30) in all models.

#### Mediating variables

Three variables recorded at the first interview after the participant’s 40^th^ birthday were assessed as potential mediators of women’s educational attainment by age 30 and self-rated health at age 50: participant income, spouse/partner income, and frequency of vigorous exercise.

*Participant income* The NLSY79 created a summary variable for each interview year representing participant’s total income for the prior calendar year. It is a composite measure of income from employment, child support payments, government assistance programs, and other sources. Participant income was represented in $10,000 units to accommodate variance maxima allowed in MPlus.

*Spouse/partner income* If a participants reported living with a spouse or partner, they reported their spouse/partner’s income each interview for the prior calendar year: “During [the prior calendar year], how much did [Spouse/partner's name] receive from wages, salary, commissions, or tips from all (other) jobs, before deductions for taxes or anything else?” Participants not living with a spouse or partner were coded as having $0 in spouse or partner income. Spouse/partner income was logged to reduce skewness.

*Frequency of vigorous exercise* Participants were age 40 between 1998 and 2006, during which time item wording for interview questions related to health behaviours changed. In 1998 and 2000, participants were asked, “How often do you participate in vigorous physical exercise or sports, such as aerobics, running, swimming, or bicycling” and responded “3 or more times per week,” “once or twice a week,” “one to three times each month,” “less than once per month,” or “never.” From 2002 to 2006, participants were asked, “How often do you do vigorous activities for at least 10 min that cause heavy sweating or large increases in breathing or heart rate?” and indicated whether the frequency they reported was per day, week, month, or year. We recoded participant responses from 1998–2006 to represent participating in vigorous exercise never = 0, less than once per month = 1, monthly = 2, weekly = 3, or daily = 4. From the 1998–2000 interviews, participants reporting participating in exercise “3 or more times per week” were recoded as “daily,” those reporting “once or twice a week” were recoded as “weekly,” and those reporting “one to three times each month” were recoded as “monthly.” From 2002 to 2006, participants reporting a frequency of vigorous exercise per year were recoded as “less than once per month.”

#### Social selection control variables

We controlled for potentially confounding variables first measured at Wave 1: race/ethnicity (Non-Hispanic/Black, Hispanic, Non-Black/non-Hispanic), participants’ mothers’ highest grade completed, Armed Forces Qualification Test (AFQT) aptitude test score percentile, educational expectations (how many years of schooling they expected to complete in their lifetime), whether the participant was enrolled in school, and participants’ number of siblings, and a self-esteem score measured at Wave 2. Race/ethnicity was included because Black mothers who give birth as teenagers report worse health at mid-life than White women who give birth as teenagers [38] as a result of persistent structural discrimination and inequality and their “weathering” effect on health [39, 40]. Self-esteem is associated with both educational attainment [41] and health at midlife [42]. The number of siblings one grows up with is predictive of educational attainment [43] and also childhood health [44], which sets the stage for midlife health [45]. In order to reduce the number of control variables included in analytic models [46], we used principal component scores for 2 retained components from a principal component analysis (PCA) of a polychoric correlation matrix [47] that together explained 61% of the variance of all social-selection control variables.

### Analysis

We estimated path models in MPlus version 7 [48]. We fit path models using maximum likelihood estimation with robust standard errors (MLR), which estimates standard errors robust to non-normality of observed data and accounts for missing data using full-information maximum likelihood (FIML). Unstandardized beta coefficients with standard errors are presented for each path. We determined model fit using multiple indices: Root Mean Square Error of Approximation (RMSEA), Comparative Fit Index (CFI), Tucker Lewis Index (TLI), and the Standardized Root Mean Square Residual (SRMR) [49]. RMSEA of < 0.06, CFI of > 0.95, TLI of > 0.95, and SRMR of < 0.08 provide evidence of good model fit [50]. We tested differences in model fit among nested models with Satorra-Bentler scaled chi-square difference tests [51] and identified the best-fitting, most parsimonious path model. We first estimated a baseline single-group model with all women (*N* = 301). In this model, all control variables were correlated with education variables, and direct paths between endogenous variables were minimal (Fig. 1). The most parsimonious baseline model demonstrated poor model fit; therefore, we estimated additional parameters, informed by both theory and the modification indices, until satisfactory model fit was achieved.

## Results

**Fig. 2:**
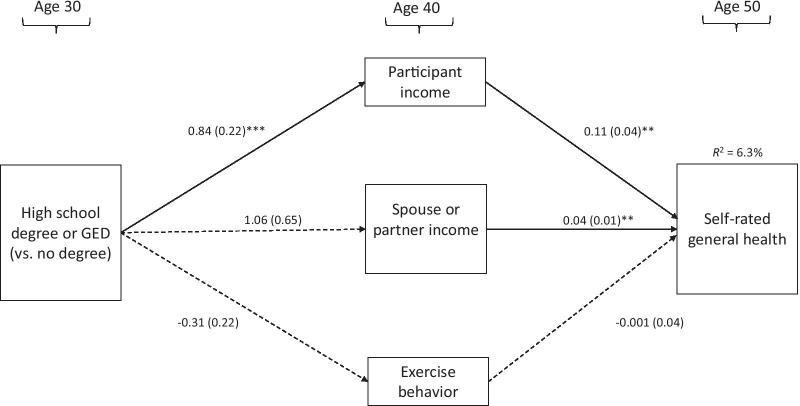
Path model of indirect paths from degree type attained by age 30 to self-rated health at age 50 (*N* = 301)

Results are depicted in Fig. 2. Correlations among control variables and paths between control variables and variables of interest are omitted from the figure for simplicity. The final model fit the data well (CFI = 1.000; TFI = 1.018; RMSEA = 0.000; SRMR = 0.035).Completing a high school degree or GED by age 30 was positively associated with respondent income at age 40; in turn, higher income at age 40 was associated with better self-rated general health at age 50. In other words, for teenage mothers, greater educational attainment was indirectly associated with better self-rated health at age 50 via increased earnings in adulthood. Tests of indirect effects are presented in Table 2. The total indirect effect from educational attainment to health at age 50 was significant. Contrary to our hypothesis, higher respondent education was not associated with higher income by their spouse/partner. However, having a higher-earning spouse/partner at age 40 was significantly associated with more positive health at age 50. In addition, higher levels of education were not associated with exercise behaviour at age 40, nor was exercise behaviour associated with better self-rated health.

**Table 2:** Indirect paths from degree type at age 30 to self-reported health at age 50

			B	SE
GED/HS→	Participant income→	Self-reported health	0.090	0.039*
GED/HS→	Spouse/partner income→	Self-reported health	0.042	0.030
GED/HS→	Exercise behavior→	Self-reported health	0.000	0.012
Total indirect effect				
GED/HS→	Self-reported health		0.133	0.048**

Unstandardized indirect path coefficients and standard errors shown

GED, General Education Development/High School Equivalency Certificate (v. no degree). HS, high school diploma (v. no degree)

*p < .05, **p < .01, ***p < .001

## Discussion

Most research to date examining the education of teen mothers assesses short-term outcomes, such as developmental and early academic outcomes of their children [52–54]. We extend this body of research by examining the association between education and teenage mothers’ health at age 50 and demonstrate that higher levels of education have long-term positive associations with health through mid-life for these women. Importantly, we examined women who had their first child before they had reached the age at which they would be expected to finish their secondary education. This allowed us to examine the association of education attained after the birth of the first child with later earnings and health. Our findings suggest that the well-established positive association between education and life-long health [12, 22] holds true for women who began childbearing early, an understudied group in this line of research. Furthermore, by disentangling educational attainment from later-life mediators, our findings add to previous research [11] by noting that that the relationship between educational attainment and self-rated health at age 50 for women with early childbearing occurs through higher annual incomes in adulthood.

As education is a social determinant of health that is amenable to intervention after a teen gives birth, these findings are supportive of higher educational attainment as a potential pathway to improving long-term health outcomes of women who begin childbearing early. Following fundamental cause theory, greater educational attainment provides access to a variety of resources, such as opportunities for employment, entrée into higher educated social networks, access to better health care, and knowledge about health-promoting behaviours, that can be deployed in pursuit of better health. This study suggests that income is one particularly salient health-promoting resource for women with a history of early childbearing.

Strengths of this study include its use of a national, prospective longitudinal sample to assess the associations of education with health over a 20-year period of women’s lives. However, the study also had several limitations. First, it was not possible to reliably assess the relationships between postsecondary education and later life health because only four women in this sample completed a four-year college degree. As higher education is becoming increasingly important for positive health, social, and economic outcomes for women and their families, there is a need to replicate this research with samples including women with early childbearing who have completed college education programs to understand the contributors to their success and its long-term impacts on their health. Second, the sample size of women was relatively small (*N* = 301) and inhibited the assessment of other, potentially salient, mediators, moderators, and background characteristics. For example, educational attainment of teenage mothers may differ by marital status at birth [55]. Furthermore, recent research suggests that women who are the least likely to experience early child-bearing face the greatest penalties regarding educational attainment and earnings [56]. Third, we were not able to examine differences in the quality of education women received, only its quantity. Higher quality education programs likely result in stronger skills and higher earning potential, but those programs may be less accessible to teenage mothers. Finally, while we adjusted models for observed background characteristics demonstrated to select women into early childbearing and lower educational attainment, findings may be attributable to unobserved factors and therefore cannot be interpreted as causal. Many programs and policies with the aim of supporting pregnant and parenting teens’ educational attainment already exist across the United States [57]. Thus, rigorous experimental studies of current and future programs and policies that support pregnant and parenting teens’ education are needed to assess the causal short and long-term effects on women’s health and well-being.

## Conclusion

Despite decades of programming focused on supporting pregnant and parenting teens, women who begin childbearing during the teenage years continue to experience relatively poor health compared to women who delay childbearing. The current study highlights the potential of educational attainment as a promising means of mitigating the poverty and poor health historically associated with teenage childbearing in the United States. What has previously been documented as a promising strategy for improving short-term outcomes for women and their children now also demonstrates promise for promoting health status into mid-life for women who began childbearing during the teenage years.

## Data Availability

The datasets used and/or analysed during the current study are available from the corresponding author on reasonable request.

## Abbreviations

AFQT: Armed Forces Qualification Test
CFI: Comparative Fit Index
FIML: Full-information maximum likelihood
GED: General Education Development/High School Equivalency Certificate
HS: High school
MLR: Maximum likelihood estimation with robust standard errors
NLSY79: National Longitudinal Survey of Youth 1979
PCA: Principal components analysis
RMSEA: Root Mean Square Error of Approximation
SRMR: Standardized Root Mean Square Residual
TLI: Tucker Lewis Index
